# Advances in Carbon Fiber Reinforced Polymers

**DOI:** 10.3390/ma19020231

**Published:** 2026-01-07

**Authors:** Francesca Lionetto

**Affiliations:** Department of Engineering for Innovation, University of Salento, Via Monteroni, 73100 Lecce, Italy; francesca.lionetto@unisalento.it

Carbon fiber reinforced polymers (CFRPs) have become increasingly widespread across a remarkable range of industries thanks to their unique combination of strength, lightness and durability [1]. These advanced composite materials are now commonly used in aerospace engineering, automotive manufacturing, marine technologies, sports equipment, sustainable construction, and even biomedical devices [2]. Their ability to withstand high stress while significantly reducing structural weight makes them ideal for applications where performance and efficiency are essential [3].

Because their use is expanding so rapidly, studying carbon fiber reinforced polymers has become an important focus of scientific research. Scientists and engineers are working to better understand the behavior of CFRPs under extreme conditions, improve manufacturing techniques, and develop new fiber–matrix combinations with enhanced mechanical and thermal properties. Research is also essential for addressing sustainability concerns, such as recycling methods and reducing the environmental impact of production.

Overall, the broad diffusion of CFRPs demonstrates their central role in modern technology and industry. Continued scientific investigation not only advances material performance but also ensures that these innovative composites can be used safely, responsibly, and efficiently in future applications.

The Special Issue Carbon Fiber Reinforced Polymers (Second Edition) gathers 7 research papers highlighting how advances in CFRPs increasingly rely on a multi-scale and multi-environment understanding of material behavior. The analysis of the published contributions in the Special Issue is reported in Table 1. From process-focused studies, such as drilling optimization and the tuning of cryogenic dynamic properties, to application-driven developments in underwater composite pipes and high-performance CFRP/steel adhesive joints, the research underscores the need to tailor interfaces and microstructures to demanding operational conditions. Parallel efforts in sustainable innovation, including vitrimer-enabled recycling routes and nonwoven fabrics derived from recycled carbon fibers, demonstrate how circularity is becoming a structural requirement in composite design. Finally, emerging manufacturing strategies, such as 3D-printed fiber–matrix interfaces, reveal how additive technologies can deliver unprecedented control over interphase architecture. Together, these directions trace a coherent roadmap for next-generation CFRPs, bridging performance, durability, manufacturability, and sustainability.

The paper of Yu et al. [4] focuses on optimizing drilling parameters for fiber-reinforced resin matrix composite laminates, which often suffer from delamination defects during processing, leading to detrimental effects on their structural integrity and mechanical properties. As reported in the literature [11,12], processing quality plays a pivotal role in determining the final performance of advanced composites. The study introduces a novel approach combining finite element simulation and experimental analysis to investigate the impact of drilling parameters on axial force and delamination. It proposes a variable parameter drilling method based on dynamic adjustment of feed rate and rotational speed, which effectively suppresses delamination propagation and improves the quality of drilled composite panels. The obtained results offer a scientific basis for improving drilling quality in laminated composite materials by mitigating delamination damage through optimized processing techniques.

In this Special Issue, beyond traditional investigations of the mechanical properties of advanced composite materials, methodological contributions emerge that focus on the characterization of interfaces in complex systems, such as the analysis of fiber–matrix interfacial dynamics in CFRP laminates under extreme service conditions (including ultra-low temperatures). The paper of Zhao et al. [5] is devoted to studying the dynamic mechanical properties and damage evolution of CFRP laminates at ultra-low temperatures, which are critical for applications in extreme environments like deep space exploration. The study combines experimental methods, theoretical modeling and numerical predictions to analyze the behavior of CFRP laminates under varying strain rates and temperatures. A numerical prediction model is developed, incorporating strain rate and temperature effects, failure criteria, and interlayer interface damage constitutive. This approach underscores how a deep understanding of interfaces with advanced techniques is crucial for the design and reliability of next-generation structural applications [13]. The obtained results provide valuable insights into how extreme environments modify viscoelastic behavior and, ultimately, influence the reliability of composite structures in extreme environments, such as lunar and Martian exploration.

The paper of Jain et al. [6] explores strategies to enhance the collapse capacity of composite cylindrical tubes for underwater applications, particularly for marine structures, such as offshore pipelines and unmanned underwater vehicles, by introducing circumferential groove geometries. These tubes are used in deep-sea environments where they must withstand high hydrostatic pressures and ensure greater collapse resistance. Composite materials are preferred over metallic structures due to their corrosion resistance, ability to operate at greater depths, and their thermal, acoustic, and magnetic insulation properties, making them ideal for underwater applications. The study combines experimental underwater implosion tests and numerical simulations using finite element modeling to investigate the effects of groove depth, steepness, and number on the structural performance of carbon fiber-reinforced composite tubes. The innovative approach demonstrates that adding grooves can increase collapse capacity by up to 20%, with deeper and steeper grooves leading to localized collapses at higher pressures. The findings provide a design framework for optimizing groove geometry and pitch distance to improve the structural resilience of underwater composite tubes under extreme hydrostatic conditions.

The paper of Okeola et al. [7] explores the use of core–shell rubber (CSR) nanoparticles to modify ambient-cured epoxy adhesives in CFRP/steel joints, aiming to improve their application in strengthening and repairing steel structures. The study addresses the challenges of adapting wet-layup CFRP systems, traditionally used for concrete, to steel substrates by enhancing adhesive properties without compromising thermal and mechanical performance. The proposed approach is able to improve the strength and durability of adhesive joints between CFRP and steel by addressing challenges related to adhesive viscosity and their adhesion ability on steel substrates. Insights into epoxy curing behavior remain highly relevant for advancing CFRP processing and performance. Understanding curing kinetics is essential to describe how the chemical reaction evolves during hardening, including reaction time and network formation [14,15]. This knowledge is crucial to optimize the mechanical and thermal properties of epoxy-based systems and to ensure reliable short- and long-term performance, particularly in structural applications such as the strengthening of steel bridges.

The paper of Pomazi et al. [8] focuses on optimizing the recycling process for polyimine-based vitrimer CFRPs. It explores the recovery of carbon fibre reinforcements and evaluates the material properties of recycled composites. The study addresses the challenges of recycling vitrimer composites, which are more sustainable and reprocessable than traditional thermoset polymers. While recycled composites showed good adhesion and mechanical properties, some residual matrix and solvent were found to impact the glass transition temperature and interlaminar shear strength. The research highlights the feasibility of recycling vitrimer composites and emphasizes the need for further optimization to ensure environmental and economic sustainability.

The paper of Nuhoglu et al. [9] focuses on investigating the fiber–matrix interface strength using a simplified single-fiber pull-out test in 3D-printed thermoset composites with Direct Ink Writing (DIW) technology. This method allows precise control of fiber alignment and eliminates the need for expensive equipment, making the testing process more accessible and cost-effective. Despite some imperfections in the specimens, the study demonstrates the feasibility of this innovative methodology, offering a versatile and affordable solution for characterizing micromechanical properties in additively manufactured composites.

In parallel with advances in composite performance, the development of in situ analytical methods is expanding interface-resolved diagnostics for materials research. These approaches highlight the central role of surface and interphase characterization in understanding and optimizing material performance and durability [16,17,18,19].

The paper of Kim et al. [10] explores the impact of carboxymethyl cellulose (CMC) and polyvinyl alcohol (PVA) on the dispersibility and chemical functional groups of wet-laid nonwoven fabrics made from recycled carbon fibers (rCFs). The study focuses on optimizing the manufacturing conditions for rCF-based nonwoven fabrics, aiming to improve their dispersibility and mechanical properties while investigating changes in chemical surface properties. The study reveals that desizing treatment significantly improves fiber dispersibility, and that the length of carbon fibers affects both dispersibility and tensile strength. The research also highlights the role of CMC and PVA in modifying oxygen-containing functional groups, enhancing surface free energy, and reducing contact angles, which contribute to improved interfacial bonding strength.

The Guest Editor is confident that this Special Issue will benefit a large community of academic and industrial researchers interested in advanced composites to further advance along the pathway toward sustainability, performance, and competitiveness.

Looking ahead, the research directions highlighted in this Special Issue suggest exciting opportunities for future developments in carbon fiber reinforced polymers. Continued progress in multi-scale process optimization, operando characterization, and advanced modelling will enable increasingly precise control over fiber–matrix interfaces, damage evolution, and long-term performance under extreme operating environments. Meanwhile, emergent sustainable pathways, including vitrimer-based reprocessability, circular feedstocks, and nonwoven architectures from recycled fibers, will help redefine the lifecycle of composite materials, shifting from end-of-life mitigation to full material circularity. Additive manufacturing and robotic deposition are expected to expand design freedom further, fostering architected interphases and highly integrated multifunctional components. Across these directions, interdisciplinary convergence between materials science, chemistry, mechanics, and digital engineering will be crucial. Together, these advances point to a future in which CFRPs are not only stronger and more reliable but also smarter, more energy-efficient, and sustainably designed for the next generation of structural applications.

## Figures and Tables

**Table 1:** Analysis of the published contributions in the Special Issue.

Contribution	Topic	Aim	Methodology
Yu et al. [4]	Drilling optimization	Reduce delamination and improve hole quality	FEM analysis; experimental analysis
Zhao et al. [5]	Cryogenic dynamic properties	Understand mechanical response in extreme environments	Dynamic testing; modeling; simulation
Jain et al. [6]	Underwater composite pipes	Increase collapse capacity in marine environments	Implosion testing; FEM analysis
Okeola et al. [7]	Adhesives for CFRP/steel joints	Improve joint strength and ductility	Epoxy modification; joint testing
Pomazi et al. [8]	Vitrimer composite recycling	Optimize fiber recovery and material reuse	Chemical dissolution; mechanical testing
Nuhoglu et al. [9]	Nonwoven fabrics from recycled carbon fibers	Improve dispersibility and mechanical properties	Effect of additives; chemical and mechanical analysis
Kim et al. [10]	3D printed fiber-matrix interface	Evaluate interface strength in additive manufacturing	Direct Ink Writing; pull-out testing
